# Association between the CTLA-4 +49A/G polymorphism and Graves’ disease: A meta-analysis

**DOI:** 10.3892/etm.2012.618

**Published:** 2012-06-20

**Authors:** XIAOYU SI, XIUFENG ZHANG, WENRU TANG, YING LUO

**Affiliations:** 1 Faculty of Environmental Science and Engineering and; 2 Laboratory of Molecular Genetics of Aging and Tumor, Faculty of Life Science and Technology, Kunming University of Science and Technology, Kunming, Yunnan 650500, P.R. China

**Keywords:** cytotoxic T-lymphocyte-associated antigen-4 gene, autoimmune thyroid diseases, Graves’ disease, meta-analysis

## Abstract

The +49A/G polymorphism of the cytotoxic T-lymphocyte-associated antigen-4 gene (CTLA-4) has been associated with Graves’ disease (GD). However, results have been inconsistent. The aim of this study was to quantitatively summarize the evidence for CTLA-4 +49A/G polymorphism and GD. Electronic search of PubMed was conducted to select studies. Case-control studies containing available genotype frequencies of CTLA-4 +49 were chosen, and Odds ratio (OR) with 95% confidence interval (CI) was used to assess the strength of this association. Forty-two case-control studies including 8,288 cases and 9,372 controls were identified. Three studies were eliminated from the total 42 studies due to a p-value <0.05 (p-value for Hardy-Weinberg equilibrium in control group) in these studies which induced significant publication bias. The overall results suggested that the variant genotypes were highly associated (p<0.01) with GD risk in all genetic models (additive model: OR, 1.443; 95% CI, 1.319–1.578; p<0.001; recessive model: OR, 1.589; 95% CI, 1.396–1.808; p<0.001; dominant model: OR, 1.621; 95% CI, 1.430–1.837; p<0.001). Similarly, in the subgroup analyses for ethnicity (Caucasian, Asian), the results were positive. This meta-analysis suggests that the CTLA-4 +49A/G polymorphism is highly associated (p<0.01) with increased risk of GD, especially in Caucasians and Asians. To validate this association, further studies with larger participants worldwide are needed to examine associations between this polymorphism and GD.

## Introduction

Graves’ disease (GD) is one of the autoimmune thyroid diseases (AITDs) which affect 5% of the general population (1). GD is an autoimmune antibody-mediated, thyroid-specific autoimmune disease which causes thyroid gland tumefaction. GD patients make antibodies to the thyroid-stimulating hormone receptor leading to hyperthyroidism. People of Western countries (∼1.2%) and 0.25–1.09% of people of China are afflicted with GD (2,3). Although environmental factors, such as infection (4) and stress, are very important in the process of Graves’ disease in susceptible individuals, one study in twins revealed that ∼80% of the predisposition to GD is due to genetic factors (5). Several genetic loci have been implicated in the susceptibility to this disease. One of the associated genes is the cytotoxic T-lymphocyte-associated antigen-4 (CTLA-4) gene which consists of 4 exons and 3 introns. In 1997, Yanagawa *et al* (6), Marron *et al* (7) and Donner *et al* (8) initially reported that there was an association between CTLA4 and Graves’ disease. The CTLA-4 gene is located on the long arm of chromosome 2q33 and belongs to the immunoglobulin superfamily. Since the CTLA-4 protein transmits an inhibitory signal to T-cells, it has a strong susceptibility in autoimmunity. One of the CTLA-4 gene polymorphisms is located on exon 1 +49, which causes a threonine to alanine substitution in codon 17 (codon 17 T/A). To date, the CTLA-4 +49A/G polymorphism has been studied in different and numerous groups in humans, and a potential association with GD has been found in many results (6–36). However, some results suggest that there is no association between CTLA-4 +49A/G polymorphism and GD (37–46). Thus, the results are still inconsistent. Another problem is that these published studies only refer to a rather modest sample size that limits their significance. Utilizing the advantage of meta-analysis, a powerful method for quantitatively summarizing different study results, we combined the data for analysis and increased the sample size to a reasonable level. In this study, we conducted a meta-analysis to quantitatively assess the effect of the CTLA-4 +49A/G polymorphism on the risk of GD.

## Materials and methods

### Publication search

PubMed was searched using the terms ‘CTLA 4’, ‘Graves’ and ‘polymorphism’ or ‘CTLA4’, ‘Graves’ and ‘polymorphism’ or ‘cytotoxic T lymphocyte’, ‘Graves’ and ‘polymorphism’ (the last search update was on March 11, 2012). Case-control studies containing available genotype frequencies of 49A/G were chosen. Additional studies were identified by a manual search of the references of the original studies.

### Statistic analysis

For the control group of each study, the observed genotype frequencies of the CTLA-4 +49A/G polymorphism were assessed for Hardy-Weinberg equilibrium using the χ^2^ test. The strength of association between the +49A/G polymorphism of the CTLA-4 gene and GD was assessed by calculating crude odds ratios (ORs) with 95% confidence intervals (CIs). The pooled ORs were performed for the additive genetic model (G vs. A), dominant model (G/G+G/A vs. A/A) and recessive model (G/G vs. G/A+A/A), respectively. Heterogeneity assumption was checked by a χ^2^-based Q-test. A p-value of <0.05 for the Q-test indicated a lack of heterogeneity among the studies; the summary OR estimate of each study was calculated by the random effects model (47,48). The potential for publication bias was examined by Begg’s test (funnel plot method) and Egger’s linear regression test (p<0.05 was considered representative of statistical significance) (49). All statistical analyses were performed with Stata software (version 11.0; Stata Corporation, College Station, TX).

## Results

### Eligible studies

We identified 42 case-control studies concerning the association between the CTLA-4 +49A/G polymorphism and GD, which included 8,288 GD cases and 9,372 controls. These data were used in our meta-analysis (Table I). The distribution of genotypes in the controls of all the studies was in agreement with Hardy-Weinberg equilibrium.

### Meta-analysis

**Figure 1:**
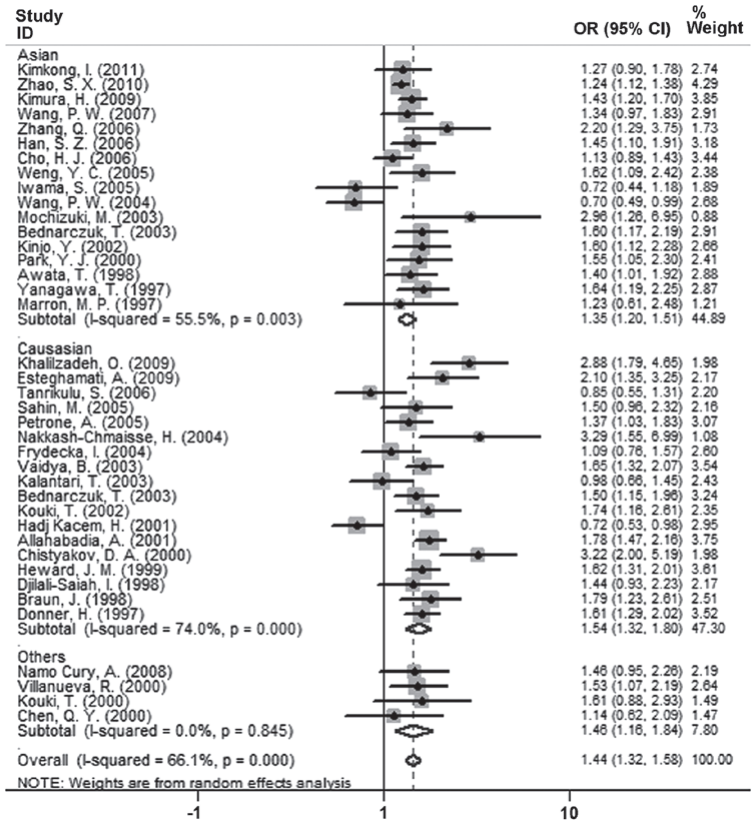
Forest plot of ORs of the G allele when compared to the A allele (additive model) in the Graves’ patients. The squares and horizontal lines correspond to the study-specific OR and 95% CI. The area of the squares reflects the study-specific weight. The diamond represents the pooled OR and 95% CI. OR, odds ratio; CI, confidence interval.

**Figure 2:**
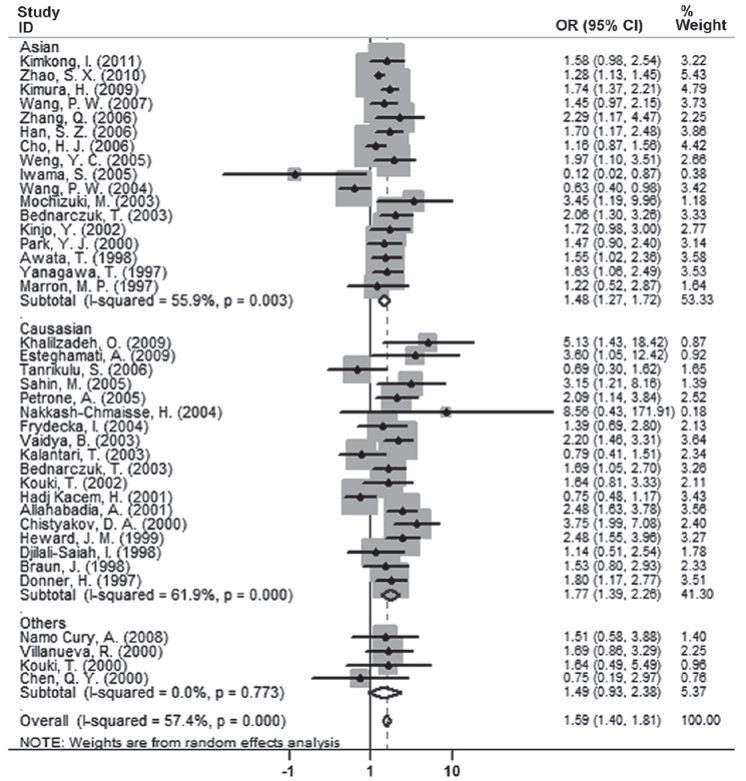
Forest plot of ORs of the G/G genotype when compared to the A allele carriers (G/A+A/A) (recessive model) in the Graves’ patients. The squares and horizontal lines correspond to the study-specific OR and 95% CI. The area of the squares reflects the study-specific weight. The diamond represents the pooled OR and 95% CI. OR, odds ratio; CI, confidence interval.

**Figure 3:**
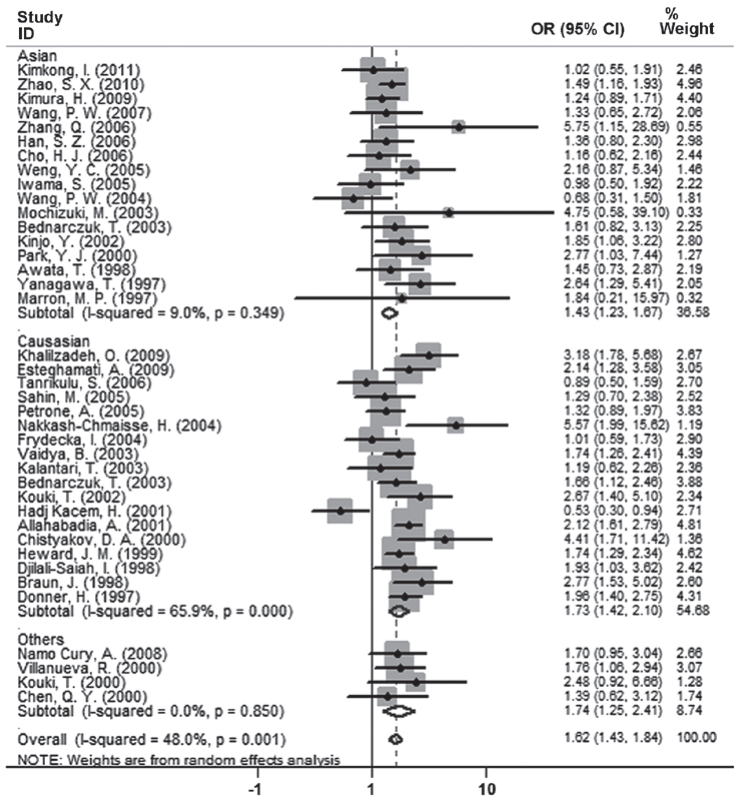
Forest plot of ORs of GD G allele carriers (G/G+G/A) when compared to the A/A genotype (dominant model) in the Graves’ patients. The squares and horizontal lines correspond to the study-specific OR and 95% CI. The area of the squares reflects the study-specific weight. The diamond represents the pooled OR and 95% CI. OR, odds ratio; CI, confidence interval.

The results of the association between the CTLA-4 +49A/G polymorphism and GD and the heterogeneity test are shown in Table II. The overall results suggest that the variant genotypes were highly associated (p<0.01) with GD risk in all genetic models [additive model: OR, 1.443; 95% CI, 1.319–1.578; p<0.001 (Fig. 1); recessive model: OR, 1.589; 95% CI, 1.396–1.808; p<0.001 (Fig. 2); dominant model: OR, 1.621; 95% CI, 1.430–1.837; p<0.001 (Fig. 3)]. Similarly, in subgroup analyses for ethnicity (Caucasians, Asians), the results were positive.

### Publication bias

Funnel plot and Egger’s test were performed to estimate the publication bias of studies. The results of Egger’s test provided statistical evidence for funnel plot symmetry (for G/G+G/A vs. A/A, p=0.166) (Table II).

## Discussion

This meta-analysis examined the association of the CTLA-4 +49A/G polymorphism with GD and included 8,288 GD cases and 9,372 controls. Three studies were eliminated from the total 42 studies due to a p-value of <0.05 (p-value for Hardy-Weinberg equilibrium in control group) in these studies which induced significant publication bias. The results of Egger’s test provided statistical evidence for funnel plot symmetry (for G/G+G/A vs. A/A, p=0.166). The overall results suggest that the variant genotypes were highly associated (p<0.01) with GD risk in all genetic models (additive model: OR, 1.443; 95% CI, 1.319–1.578; p<0.001; recessive model: OR, 1.589; 95% CI, 1.396–1.808; p<0.001; dominant model: OR, 1.621; 95% CI, 1.430–1.837; p<0.001). Similarly, in subgroup analyses for ethnicity (Caucasians, Asians), the results were positive.

GD is a disease with significant clinical consequences. The mechanism of GD is still relatively unknown. Although environmental factors, such as infection (4) and stress, are important in the process of Graves’ disease in susceptible individuals, one study in twins suggests that ∼80% of the predisposition to GD is due to genetic factors (5). Single nucleotide polymorphisms (SNPs) can be used as a tool for investigating genetic variations and disease susceptibility. GD is an autoimmune antibody-mediated, thyroid-specific autoimmune disease. The CTLA-4 protein can transmit an inhibitory signal to T-cells and has a strong susceptibility in autoimmunity. CTLA-4 protein has recently been described as a gatekeeper of conjugation timing and reduced conjugation may protect against prolonged contact periods of cytotoxic T lymphocytes with autoantigen-defined targets (50). It has been in the centre of attention for its key role in autoimmunity. The +49A/G polymorphism is one of the CTLA-4 three forms of polymorphisms. To date, a multitude of different studies were carried out concerning the association between the CTLA-4 +49A/G polymorphism and GD, but the results are inconsistent. In many studies (6–36) the results are positive, however in others (37–46) the results are negative.

This meta-analysis revealed a highly significant (p<0.01) association between the CTLA-4 +49A/G polymorphism and GD risk, in both Asian and Caucasian subgroups. In conclusion, this meta-analysis suggests that the CTLA-4 +49A/G polymorphism is potentially associated with the risk of GD among Caucasians and Asians. Future, well-designed, large scale studies are necessary to validate this association in different populations.

## Figures and Tables

**Table I:** Distribution of the CTLA-4 +49A/G genotype for patients with Graves’ disease and the controls.

Population	Ethnicity or origin	Study		GD			Control			
			Year	A/A	A/G	G/G	A/A	A/G	G/G	P-value^a^
Caucasian	South Indian	Veeramuthumari *et al* (9)	2011	11	37	32	29	25	26	0.000819
Asian	Thai	Kimkong *et al* (37)	2011	22	49	61	26	73	54	0.875319
Asian	Chinese Han	Zhao *et al* (10)	2010	104	730	1030	156	823	945	0.211832
Asian	Japanese	Kimura *et al* (11)	2009	62	143	210	142	358	295	0.067982
Caucasian	Iranian	Khalilzadeh *et al* (12)	2009	48	43	14	75	25	3	0.606930
Caucasian	Iranian	Esteghamati *et al* (13)	2009	114	71	20	75	25	3	0.606930
Others	Brazilian	Namo Cury *et al* (38)	2008	43	58	15	39	32	7	0.905523
Asian	Chinese	Chong *et al* (14)	2008	7	73	97	24	56	71	0.028090
Asian	Taiwanese	Wang *et al* (15)	2007	15	69	124	18	77	97	0.633099
Asian	Chinese	Zhang *et al* (16)	2006	2	29	58	7	26	27	0.846451
Caucasian	Turkish	Tanrikulu *et al* (17)	2006	48	38	11	42	34	14	0.120930
Asian	Chinese	Han *et al* (18)	2006	33	95	135	32	89	75	0.520341
Asian	Korean	Cho *et al* (39)	2006	16	112	160	30	197	244	0.240107
Asian	Taiwanese	Weng *et al* (40)	2005	8	53	46	15	58	28	0.091603
Caucasian	Turkish	Sahin *et al* (19)	2005	29	33	15	43	48	7	0.189953
Caucasian	Italian	Petrone *et al* (20)	2005	59	68	23	139	138	24	0.201228
Asian	Japanese	Iwama *et al* (41)	2005	17	25	1	78	88	34	0.287293
Asian	Taiwanese	Wang *et al* (21)	2004	18	72	81	11	50	87	0.316477
Caucasian	Lebanese	Nakkash-Chmaisse *et al* (22)	2004	8	23	3	24	14	0	0.163933
Caucasian	Polish	Frydecka *et al* (42)	2004	32	50	17	50	84	20	0.096480
Caucasian	White	Vaidya *et al* (23)	2003	88	139	74	146	158	45	0.825642
Asian	Japanese	Mochizuki *et al* (24)	2003	1	6	13	12	27	21	0.539129
Caucasian	Iranian	Kalantari *et al* (25)	2003	21	49	20	30	53	30	0.510214
Caucasian	Polish	Bednarczuk *et al* (26)	2003	75	123	66	77	85	32	0.303455
Asian	Japanese	Bednarczuk *et al* (26)	2003	28	140	151	15	63	34	0.093804
Asian	Chinese	Yung *et al* (27)	2002	3	54	66	23	59	76	0.046372
Caucasian	USA	Kouki *et al* (28)	2002	22	67	31	30	36	14	0.576150
Asian	Japanese	Kinjo *et al* (32)	2002	32	62	50	38	46	26	0.107271
Caucasian	Tunisian	Hadj Kacem *et al* (30)	2001	31	63	50	26	94	85	0.998814
Caucasian	UK Caucasian	Allahabadia *et al* (43)	2001	136	262	86	192	198	34	0.081624
Others	African, American, Hispanic, Asian	Villanueva *et al* (44)	2000	42	67	28	53	52	16	0.568526
Asian	Korean	Park *et al* (31)	2000	5	35	57	26	75	98	0.061219
Others	Not specified	Kouki *et al* (32)	2000	8	29	8	15	23	5	0.390573
Caucasian	Moscow	Chistyakov *et al* (33)	2000	6	22	50	25	38	30	0.081864
Others	African-American	Chen *et al* (45)	2000	20	25	4	23	19	5	0.718804
Caucasian	UK	Heward *et al* (34)	1999	122	192	65	164	171	28	0.067423
Caucasian	White	Djilali-Saiah *et al* (46)	1998	23	37	13	47	37	16	0.069793
Caucasian	German, Canadian	Braun *et al* (35)	1998	22	56	25	52	48	21	0.096985
Asian	Japanese	Awata *et al* (36)	1998	11	44	57	58	197	170	0.938310
Asian	Japanese	Yanagawa *et al* (6)	1997	11	64	78	34	88	78	0.287293
Asian	Chinese	Marron *et al* (7)	1997	1	11	16	6	39	49	0.632129
Caucasian	German, Canadian	Donner *et al* (8)	1997	81	161	63	135	149	41	0.990935

a p-value for Hardy-Weinberg equilibrium in the control group. GD, Graves’ disease.

**Table II:** ORs and 95% CI for the CTLA-4 +49A/G polymorphism for different genetic models in patients with Graves’ disease.

Genetic model	Population	Pooled OR	(95% CI)	P-value	Heterogeneity P-value	Begg’s test P-value	Egger’s test P-value
Additive (G vs. A)	Asian	1.347	(1.203–1.507)	<0.001	0.003	0.323	0.373
	Caucasian	1.543	(1.324–1.798)	<0.001	<0.001	0.426	0.788
	Others	1.458	(1.157–1.837)	0.001	0.845	0.174	0.505
	Overall	1.443	(1.319–1.578)	<0.001	<0.001	0.255	0.642
Recessive (G/G vs. A carriers)	Asian	1.476	(1.267–1.721)	<0.001	0.003	0.621	0.506
	Caucasian	1.770	(1.386–2.260)	<0.001	<0.001	0.791	0.586
	Others	1.487	(0.931–2.376)	0.097	0.773	0.174	0.275
	Overall	1.589	(1.396–1.808)	<0.001	<0.001	0.978	0.965
Dominant (G carriers vs. A/A)	Asian	1.431	(1.227–1.670)	<0.001	0.349	0.187	0.196
	Caucasian	1.727	(1.419–2.102)	<0.001	<0.001	0.344	0.860
	Others	1.739	(1.254–2.412)	0.001	0.850	1.000	0.705
	Overall	1.621	(1.430–1.837)	<0.001	0.001	0.113	0.166
